# The health effects of menthol cigarettes as compared to non-menthol cigarettes

**DOI:** 10.1186/1617-9625-9-S1-S7

**Published:** 2011-05-23

**Authors:** Allison C Hoffman

**Affiliations:** 1 Center for Tobacco Products, Food and Drug Administration, Rockville, MD 20850, USA

## Abstract

Since the 1920s, menthol has been added to cigarettes and used as a characterizing flavor. The health effects of cigarette smoking are well documented, however the health effects of menthol cigarettes as compared to non-menthol cigarettes is less well studied. This review discusses menthol’s effects on 1) biomarkers of tobacco smoke exposure, 2) toxicity and cellular effects, 3) lung function and respiration, 4) pulmonary and/or vascular function, 5) allergic reactions and inflammation, and 6) tobacco-related diseases. It is concluded that menthol is a biologically active compound that has effects by itself and in conjunction with nicotine, however much of the data on the other areas of interest are inconclusive and firm conclusions cannot be drawn.

## Introduction

Tobacco use produces a myriad of negative health effects, and has caused more than 5 million premature deaths through diseases such as cancer, cardiovascular disease, and respiratory disease [1]. Given the large number of menthol cigarette users (nearly 27% of current smokers, with Black/African American smokers being about 3 times more likely to smoke menthol cigarettes as compared to white smokers [2,3]) there is less information on the health of effects of menthol cigarettes as compared to non-menthol cigarettes than might be expected. Indeed, although there are many research articles that point to menthol as a possible contributing factor to several health effects of smoking, the majority did not investigate menthol as an independent factor and instead focused on racial/ethnic disparities on use. This review seeks to explore the available literature on the health effects of menthol cigarettes as compared to non-menthol cigarettes. Questions addressed in this review include:

• What, if any, are menthol’s effects on biomarkers of tobacco smoke exposure?

• What effect, if any, does menthol have on a cellular level?

• What effect, if any, does menthol have on respiration?

• What effect, if any, does menthol have on cardiovascular function?

• What allergic reactions have been associated with menthol cigarettes?

• What effect, if any, does menthol have on smoking-related disease?

Summarized in this review are 89 articles found to have either direct relevance to these questions, or were used to provide relevant background information. Many of these articles were identified through a review of the literature conducted by the National Cancer Institute in 2009, published as “Bibliography of literature on menthol and tobacco” http://cancercontrol.cancer.gov/tcrb/documents/menthol_bibliography_508.pdf. Search terms used were menthol cigarette(s); mentholated cigarette(s); menthol tobacco; mentholated tobacco; menthol smoker(s); menthol AND the following terms: addiction, nicotine, marketing, cancer, biomarkers, asthma, cardiovascular disease, heart disease, vascular disease, chronic obstructive lung disease, respiratory, environmental tobacco smoke, national health, health disparities, and minority health. Additional searches and sources, such as those identified through review articles, identified additional articles that were included as appropriate. A publication or study is identified as having a tobacco industry association if one or more authors were employees of the tobacco industry, as identified by author affiliation on the publication.

Of those articles that are in the NCI Bibliography but were not included, most were not directly relevant to this topic (e.g., they studied menthol as a chemical independent from tobacco smoke exposure, did not evaluate menthol as a separate variable). Some of those articles, however, were used to provide background information. Animal or in vitro research was included only to help explain human findings. Although a few review articles were used to make general statements and/or provide background information, most were not included in deference to original sources. Published abstracts were not included out of concern that, due to the lack of details, those studies could not adequately be assessed.

## Biomarkers of tobacco smoke exposure in menthol and nonmenthol smokers

### Nicotine

Inhalation of drugs is a very effective means of delivery because inhaled drugs avoid first-pass metabolism by the liver, which rapidly metabolizes nicotine through the enzyme cytochrome P450 (CYP2A6). The main metabolite of nicotine is cotinine, and cotinine concentrations in blood, urine, or saliva are often used as biomarkers to evaluate tobacco use and exposure to environmental tobacco smoke [4-6]. Cotinine has a longer half-life (average 18-20 hours) than does nicotine (2-3 hours) [5], making it a more feasible marker of tobacco use than nicotine. Cotinine is further metabolized to trans-3’-hydroxycotinine (3 HC). Although glucuronidation (a process by which glucuronic acid is conjugated with a substrate) is usually a minor metabolic pathway for nicotine and cotinine, it can be a major determinant of nicotine clearance in people who have low CYP2A6 activity. That is, in people who metabolize nicotine more slowly through the CYP2A6 pathway, the glucuronidation pathway may metabolize a larger share of nicotine. Thus, glucuronidation may become a more significant factor in overall nicotine metabolism. Slower metabolism of nicotine means that levels of nicotine in the body remain elevated for a longer period of time, allowing a longer timeframe for nicotine to interact with nicotinic receptors all over the body.

The authors of a small cross-over study of seven Black/African American and seven White smokers found that menthol cigarette smoking resulted in slower nicotine metabolism and slower total nicotine clearance. Overall, there were no significant racial/ethnic differences in the disposition kinetics of nicotine [7]. Menthol cigarette smoking was associated with reduced nicotine metabolism through a decrease in CYP2A6 enzyme activity and a substantial reduction in glucuronidation. These findings were supported in an in vitro study using human microsomes that reported that menthol inhibits the CYP2A6 enzyme, resulting in inhibition of nicotine metabolism [8].

Using data from a study of 755 Black/African American smokers who smoked fewer than 10 cigarettes a day (so-called “light” smokers), Ho and colleagues [9] found that smokers who smoke menthol cigarettes had slower metabolism of cotinine as compared to non-menthol smokers.

Clark et al [10] studied the effects of menthol cigarettes on biochemical markers of smoke exposure among 161 adult Black/African American and White smokers in a cross-sectional study. There were also racial/ethnic differences, with African American smokers having significantly higher cotinine per cigarette ratios, but it is unknown if this is due to differences in metabolism, smoking behavior, or other reasons. After adjusting for race, cigarettes per day and average amount of each cigarette smoked, serum cotinine levels were significantly higher among menthol cigarette smokers than among smokers of non-menthol cigarettes, suggesting greater exposure to nicotine. [10] Numerous other reports [4,10-15] have found that Black/African American smokers are more likely than White smokers to smoke fewer cigarettes per day (CPD) yet have substantially higher cotinine levels. Because Black/African American individuals are considerably more likely to smoke menthol cigarettes, menthol may have been a mitigating factor; however, the potential impact of menthol was not evaluated separately in these studies.

In a between-subjects study of 95 female adult smokers stratified by race/ethnicity and menthol/non-menthol cigarette preference, Ahijevych and Parsley [11] reported that smokers of menthol cigarettes had higher cotinine levels. This finding would be expected, given menthol’s inhibition of CYP2A6 metabolism and glucuronidation [8,16,17]. As was found with Clark et al [10], Black/African American subjects had higher cotinine per cigarette levels, suggesting greater exposure to nicotine.

Data from the National Health and Nutrition Examination Survey (NHANES) were used to compare the serum cotinine levels of more than 1,500 smokers. The data from this large, nationally representative sample compared menthol and non-menthol smokers. Menthol smokers were found to have significantly higher serum cotinine levels (1333.8 ± 40.1 nmol/L) as compared to non-menthol smokers (1230.3 ± 24.5 nmol/L) [6].

In an inpatient research study by Ahijevych and colleagues [4], plasma samples were taken while the subjects were smoking as desired, followed by several days of smoking abstinence. Cotinine half-life and did not significantly differ between menthol (23.1 ± 7.9 hours) and non-menthol smokers (18.1 ± 8.1 hours) [4]. However, as has been discussed previously [10,11], there was a main effect for race, with Black/African American smokers having greater cotinine levels as compared to white smokers. Again, this suggests that these people had greater exposure to nicotine on a per cigarette basis.

A study by Patterson et al (2003) of 190 treatment-seeking smokers (29 menthol smokers, 161 non-menthol smokers) failed to find significant differences in nicotine boost (an individualized measure of how much nicotine has been extracted from smoking a cigarette) produced following the smoking a preferred-brand cigarette (p < 0.10). However, the authors note that “the absence of racial differences in boost and a lack of association with cigarette characteristics (e.g., menthol) may be attributable to the relatively small number of African Americans in the sample” [18]. Consistent with other studies [4,10,11], being Black/African American was significantly associated with greater levels of blood nicotine following a single cigarette. [18]

Mustonen et al [19] investigated possible associations between cotinine/CPD ratios in subgroups varying by gender and race/ethnicity in a randomized clinical trial. Consistent with other studies [4,10,11,18], Black/African American smokers smoked fewer CPD, but did not have significantly lower cotinine levels, suggesting a higher cotinine per cigarette ratio. Although there was a pattern toward higher cotinine levels in smokers of menthol cigarettes, it was not statistically significant. There was, however, a significant gender by race by menthol interaction on salivary cotinine level as well as cotinine/CPD ratio. Black/African American menthol cigarette smokers and Black/African American non-menthol cigarette smoking women had higher cotinine/CPD ratios than did White smokers. These findings suggest that the relationship between number of cigarettes consumed and salivary cotinine is complex. This study was limited in that puffing rate, depth of inhalation and length of cigarette smoked could not be controlled for in the study sample. The authors concluded that type of cigarette, race/ethnicity, and gender need to be evaluated concurrently [19].

Wang et al [20] investigated the effects of menthol cigarettes on adult smokers’ exposure to nicotine in a large, cross-sectional study. The menthol cigarette smokers were more likely to be Black/African American and more likely to be female, which is consistent with the demographics in other studies (for review, see Ahijevych and Garrett 2004 [21]). There were no significant differences in nicotine equivalents (nicotine and five major nicotine metabolites) per cigarette, leading the researchers to conclude that smoking menthol cigarettes does not increase daily exposure to smoke constituents. No significant differences were found in serum cotinine levels between menthol and non-menthol cigarette smokers, suggesting that menthol had no effect on the metabolism of nicotine in the study. Consistent with other studies [4,10,11,18,19], although the Black/African American smokers smoked fewer cigarettes per day, there was no different in serum cotinine levels, suggesting a higher nicotine/cigarette ratio. One limitation of the study was that only a small proportion of the Black/African American smokers smoked non-menthol cigarettes.

In a tobacco industry-associated parallel arm study designed to investigate whether moderately heavy (≥ 15 CPD) smokers of menthol cigarettes had different biomarker levels than non-menthol cigarette smokers, the researchers failed to find significant differences in urine levels of nicotine or glucuronidated nicotine metabolites. Unlike other studies [4,10,11,18,19], they found no significant racial/ethnic differences in metabolism [22]. A limitation of the study was the small number of Black/African American non-menthol cigarette smokers compared with the number of White non-menthol cigarette smokers.

Using a stored sample from 255 current smokers from the Southern Community Cohort Study (65 Black/African American men, 65 Black/African American women, 63 White men, 62 White women), comparisons of serum cotinine levels of menthol and non-menthol smokers were made. There were significant interactions between gender and race/ethnicity, but no significant differences were found between menthol and non-menthol groups [23]. Consistent with previous studies [4,10,11,18,19], after adjustment for CPD differences, Black/African American smokers had higher cotinine levels as compared to white smokers.

In a study of more than 700 Black/African American light smokers (≤ 5 CPD), menthol smokers did not differ in plasma cotinine levels when compared to their non-menthol smoking counterparts. This may be due to a wide range between the minimum and maximum plasma cotinine levels across smokers in the study: 5.0 ng/mL versus 937.8 ng/mL [9].

A study by Williams and colleagues [24] compared cotinine levels of menthol- and non-menthol-smoking patients with and without schizophrenia. The laboratory study of 142 people assessed blood cotinine levels during a typical smoking day, two minutes after smoking a usual-brand cigarette. There were no significant differences when comparing the schizophrenic smokers with non-schizophrenic smokers. However, menthol smokers had significantly higher serum cotinine levels as compared to non-menthol smokers (294.3 ng/ml and 238.8 mg/ml, respectively; p = .041). Menthol smokers also had significantly higher serum nicotine levels (27.2 mg/ml and 22.4 mg/ml, respectively; p = 0.01) an effect that appears to be driven by schizophrenic smokers having significantly higher serum nicotine levels as compared to non-schizophrenic smokers (p < 0.05). The authors suggest that the elevated levels observed in menthol smokers may be due in part to increased intake of smoke or menthol-mediated inhibition of nicotine metabolism [24].

### Carbon monoxide

Like the nicotine/cotinine data, the data from studies measuring expired air carbon monoxide (CO) and/or levels of blood carboxyhemoglobin (a measure of CO exposure), which are often used as biomarkers to indicate level of exposure to tobacco smoke, are not consistent. Some investigators have found that menthol cigarette smoking increased CO (as measured by expired CO, CO boost, or blood carboxyhemoglobin) as compared to non-menthol cigarettes smoke [10,24-26], whereas other studies, including one associated with the tobacco industry, have found either no difference in CO exposure [22,26-29], or a lower level among menthol smokers [30,31]. Ahijevych et al (1996) that found that menthol smokers had lower CO measurements as compared to non-menthol smokers, reporting statistically significant differences for the Black/African American menthol participants of this women-only study [30]. Possible reasons for the inconsistency of the findings include the possibility that physiologic variables, such as mucous layers in mucosal cold nerve endings or differences in how the cigarette burns (menthol pyrolysis) may affect CO levels [30,32]. Also, menthol’s effects on biomarkers such as blood carboxyhemoglobin and cotinine are not linear and, as has been noted by publicly available internal tobacco industry documents, are affected by other chemicals in the smoke [33].

### Tobacco-specific nitrosamines

Toxins present in tobacco, such as tobacco-specific nitrosamines (TSNAs), which are known carcinogens, are also used as biomarkers of tobacco smoke exposure. Menthol may alter glucuronidation metabolism of some TSNAs, such as the tobacco carcinogen, 4-(methylnitrosamino)-1-(3-pyridyl)-1-butanone; (NNAL), which goes through a glucuronic metabolic pathway to form NNAL-Glucuronide (NNAL-Gluc) [16]. Thus, inhibition of the glucuronidation process may result in adverse effects, such as an accumulation of NNAL.

As a parallel to the findings by MacDougall et al [8] in an experimental *in vitro* study, Muscat et al [16] found that menthol inhibited glucuronidation of NNAL in human microsomes; however, in an *in vivo* study of rats treated with 4-(methylnitrosamino)-1-(3-pyridyl)-1-butanone, or NNK (a TSNA that is metabolized into the TSNA NNAL), those that received oral menthol showed increased levels of NNAL metabolites. This suggests enhanced metabolism. Orally administered menthol delivered in the absence of the other constituents of tobacco smoke, as well as generalizing from rats to humans, makes generalizations regarding the possible effects of menthol tobacco smoke difficult.

In an *in vivo* component of the Muscat et al [17] study mentioned above, urinary ratios of NNAL/NNAL-Gluc in adult smokers were measured. Smokers of menthol cigarettes had lower urinary ratios of NNAL/NNAL-Gluc than smokers of non-menthol cigarettes, suggesting that menthol inhibited NNAL glucuronidation [17]. Although these findings provide additional support that menthol generally inhibits glururonidation [7,8] a tobacco industry associated study failed to find any inhibition of glucuronidation of NNAL [22]. This parallel-arm study, which measured levels of total NNAL and NNAL-gluc in the urine of moderately heavy (≥ 15 CPD) smokers of “light” cigarettes (7–15 mg Federal Trade Commission [FTC] “tar”), failed to find significant differences when comparing levels in menthol versus non-menthol smokers [21]. Differences in urinary metabolites of NNAL between Black/African American and White smokers have been found in other studies, but menthol was not specifically investigated as the cause of these differences [34].

## Toxicity and cellular effects

A tobacco industry associated study exposed rats to menthol or non-menthol cigarette smoke via nose-inhalation for 1 hour a day, five days per week for 13 weeks. Exposure to either type of cigarette smoke produced reduced body weights and histopathological changes, including epithelial hyperplasia and/or squamous metaplasia in the nasal passages, trachea and larynx, and lungs and bronchi. Olfactory epithelial degeneration was also observed. There were no differences in these changes between the types of cigarette smoke. The only difference noted between the two groups of rats was that the non-menthol tobacco smoke–exposed rats had a dose-related increase in nasal discharge [35].

Menthol appears to alter cell membranes, and the findings of animal studies have suggested, according to some, that these changes in cell membrane integrity may result in an increased potential for tobacco-related disease [36]. In a study evaluating tobacco smoke effects on transepithelial electrical resistance (TER; the tight gap junctions between the human bronchial epithelial cells), both non-menthol and menthol smoke reduced TER. This indicates that the gap junctions between the cells were “loosened” up and integrity was lost, which suggests that the smoke irritated these cells. Menthol did not appear alter this effect of tobacco smoke [37]. It does appear, however, that menthol alters cells’ permeability. Porcine esophageal tissue bathed in a solution containing menthol and NNK had a markedly lower permeation rate for NNK, and produced an increase in tissue reservoir formation. This resulted in significantly more NNK bound within the esophageal mucosa, possibly increasing cell exposure to NNK, which is something that the authors suggest may increase the likelihood of cancer of the esophagus [36]. Recently, menthol has been shown to increase permeation of both the tobacco carcinogen nitrosonornicotine (NNN) and nicotine across porcine buccal mucosa and floor of the mouth mucosa [39], which suggests menthol could increase exposure to NNN and nicotine. It should be noted that epidemiological studies on menthol cigarettes and cancer risk do not support the proposition that these cigarettes confer a risk for cancer above that of non-menthol cigarettes (see “Tobacco-Related Disease”).

One of the more immediate cellular effects of menthol on cell membranes is that of cell death (cytotoxicity). Menthol has been shown to be toxic in *in vitro* biologic model systems in normal tissue: it inhibits fatty acid-induced (receptor-mediated) cell respiration in brown adipose tissue and increased cellular respiration rate and osmotic swelling (suggesting deterioration of biologic membranes) in mitochondria [39]. In a variety of cancer cell cultures, including gastric SNU-5 cells, melanoma, myeloma, liver epithelial, neuroendocrine, bladder, and leukemia cells, as well as cells associated with prostate cancer and neuroblasoma cells, menthol dose- and time-dependently inhibits cell proliferation and/or induces cell death [40-51]. Although there is much evidence suggesting toxicity, it is important to note that this is not a universal finding (see Gordon et al 1982 [52]). Despite indications of menthol-induced cytotoxicity in both normal and diseased cells, when added to cigarettes, menthol does not appear to enhance the cytotoxicity already produced by tobacco smoke exposure. According to five tobacco industry associated research studies, both menthol and non-menthol cigarette smoke have similar levels of cytoxicity, based on results of short-term genotoxicity assays [53-57].

## Lung function and respiration

On two separate days, 74 smokers (18 menthol smokers, 56 non-menthol smokers) participating in an in-patient study were allowed to smoke one of their usual brand of cigarettes. During this time, their breathing patterns were measured. This tobacco industry associated study found that the average inhalation tidal ratio (a measure of lung volume) was 1.52 for menthol smokers and 1.79 for the non-menthol smokers, which suggested menthol smokers had lower inhalation tidal ratios (p = 0.054). Mean inhalation volume for menthol smokers was 753 mL, which was lower than for non-menthol smokers, but this difference was not statistically significant (p = 0.11). Total lung exposure times did not significantly differ between menthol and non-menthol smokers (4.0 sec and 4.1 sec, respectively; p = 0.85) [58].

Smokers with mild-to-moderate chronic obstructive pulmonary disease were recruited into the longitudinal Lung Health Study. A total of 5,886 smokers participated in this prospective, randomized smoking cessation trial. Although demographic and population information did not include information on the number of smokers who smoked menthol cigarettes or non-menthol cigarettes, it was reported that smoking menthol cigarettes did not significantly affect the rate of decline in lung function in Year 1 (p = 0.229) or between Year 1 and Year 5 (p = 0.64), as measured by spirometry, a modified ATS-DLD-78 Respiratory Symptoms Questionnaire, and a calculated methacholine reactivity score (based on forced expiratory volume). No other comparisons of menthol and non-menthol smokers were reported [59].

According to a published analysis of publicly available internal tobacco industry documents, when added to cigarettes, menthol enables deeper inhalation and may alter the frequency or volume of inhalation patterns [60]. Most studies, however, have failed to find any effects of menthol on respiration (e.g., breathing patterns, nasal resistance). However, despite a lack of physiological effects, inhaled menthol vapor has been associated with reduced ratings of respiratory discomfort [61-63]. The dichotomy between sensation and physiological response has been noted by the tobacco industry, which (according to one published analysis of publicly available internal tobacco industry documents) has concluded that menthol increases the perception of nasal openness in the absence of actual changes in nasal resistance [60].

## Cardiovascular function

Data collected in the longitudinal Coronary Artery Risk Development in Young Adults (CARDIA) study was used to prospectively evaluate the effects of cigarette smoking on atherosclerosis and pulmonary function. This large scale, 15-year study of Black/African American and White Americans found that menthol and non-menthol cigarettes were equally harmful. Smokers of either type of cigarette had increased prevalence of coronary calcification and reduced pulmonary function [64].

In a rapid-smoking study, there was one racial/ethnic difference in the cardiovascular response of menthol versus non-menthol smokers in response to exposure to menthol cigarette smoke. In this repeated-measures laboratory study of 28 smokers, Black/African American menthol smokers had lower increases in heart rate following inhalation of menthol cigarette smoke as compared to Black/African American non-menthol smokers (4.4% increase and 12.2% increase, respectively). The increases in heart rate of Black/African American menthol and non-menthol smokers did not significantly differ following inhalation of non-menthol cigarette smoke [65].

Three laboratory cross-over studies by Ciftci and colleagues assessed various cardiovascular outcomes following the smoking of two test cigarettes (menthol or non-menthol). Following the smoking of menthol cigarettes, there were generally worse cardiovascular outcomes, including worse ventricular diastolic function [66], greater increases in heart rate (101.2 bpm compared to 83 bpm), greater increase in systolic blood pressure (130.7 mmHG compared to 118.0 mmHg), and greater stiffness of the carotid artery (stiffness index of 5.7 compared to 2.2) [67]; however, there was no difference on measures of coronary flow reserve [68]. It is not known whether the participants were usually menthol or non-menthol smokers.

In a small cross-over laboratory study (n = 22), the effects of “denicotinized” test menthol and non-menthol cigarettes on a range of psychophysiologic and subjective variables were measured in both menthol and non-menthol smokers. No differences between the groups were found with respect to most of the variables, although menthol smokers were found to have a greater increase in heart rate after smoking the test cigarettes as compared to non-menthol smokers. Menthol smoke itself did not have any effects that differed from non-menthol smoke; however, menthol smokers had greater increases in heart rate, regardless of test cigarette, as compared to non-menthol smokers [69]. This appears to be a smoker difference rather than a menthol versus non-menthol cigarette difference. Since the nicotine had been removed from the test denicotinized menthol and non-menthol cigarettes, this suggests that menthol smokers may be more sensitive to non-nicotinic, and even non-menthol, components of smoking, such as other chemical components or sensory cues.

## Tobacco-related disease

### Basic research

Basic research has not found evidence that menthol, by itself, causes cancer or is mutagenic [70-74]. It may, however, affect cancers induced by other chemicals. Three animal studies investigated the effects of menthol on cancer. A tobacco industry associated study found that menthol cigarette smoke condensate painted on mouse skin did not significantly alter tumor incidence, latency, or multiplicity as compared to non-menthol cigarette smoke condensate. Both menthol and non-menthol cigarette smoke condensate produced 3–11 tumors per mouse [75]. A second study examined the effect of orally administered menthol on cancer of the large bowel and duodenum in rats, and found that menthol did not significantly alter the percent of rats with tumors (50% of control rats, 42% of menthol-treated rats ) or number of tumors per rat (1.5 tumors in control rats and 1.2 tumors in menthol-treated rats) [76]. A third study also examined the effect of orally administered menthol on carcinogenesis. In a rat mammary carcinogenesis model, rats treated with orally administered menthol had fewer average number of tumors per rat (2.0 as compared with 3.3), as well as longer median tumor latencies (80 days as compared with 63 days). These data suggest that menthol inhibited carcinogenesis and acted as a chemopreventive agent that extended tumor latency [77]. As has been previously discussed, generalization is difficult, as orally administered menthol differs greatly from menthol inhaled as a constituent of tobacco smoke.

### Clinical and epidemiological research

Possible interactions between menthol and smoking-related disease, either as a disease state or on the cellular level, have been studied. The data do not suggest that smoking menthol cigarettes is associated with an altered likelihood of developing cancer. Several studies have failed to find that menthol cigarette smoking alters the likelihood of developing several kinds of cancers, including lung and non-lung smoking related cancers, as well as cardiovascular disease or coronary heart disease [78-80] in the population as a whole.

Although some studies specifically discussed the absence of a menthol x gender x disease interaction [77,80], other studies have suggested that such an interaction may exist. One prospective study of the health of smokers found that male (but not female) menthol smokers had a modestly increased risk of lung cancer, with a relative risk of 1.45 (95% CI = 1.03–2.02) [82]. Another case control study suggested a small positive association between pharyngeal cancer in menthol-smoking males, but not females (OR = 1.7; 95% CI = 0.8–3.4), but this difference was not statistically significant [84]. A third case control study found that menthol use was not associated with changes in risk for esophageal cancer in males, but suggested that females may have a modestly increased risk (OR = 2.3; 95% CI = 0.93–5.72). These differences also failed to reach statistical significance [85]. A fourth case control study suggested that menthol may modestly increase risk of lung cancer in men with histories of more than 32 pack years of smoking menthol cigarettes (OR = 1.48; 95% CI = 0.71–3.05), but the findings were not statistically significant and limited by the small study size [86].

Many studies have compared Black/African American smokers with White smokers, finding consistently that the Black/African American smokers are at higher risk for tobacco-related disease such as lung cancer [87]. These studies, however, failed to separately assess menthol as a contributing factor, so although menthol was discussed as a possible mitigating factor, no conclusions could be drawn. Although not discussing menthol per se, an article by Etzel and colleagues [88] suggested that the lung cancer risk prevention model that is generally used may not be appropriate for Black/African American populations, since the models were developed in White populations. This is an interesting concept that is included here for discussion purposes, since menthol preference is more common among Black/African American smokers as compared to White smokers. Etzel et al developed a multivariate model of predicting risk of lung cancer in the black population. Using their model, they state that “mentholated cigarettes seemed to be protective in current smokers” [88]; however, this finding was not statistically significant [88].

## Summary

Menthol is a biologically active compound that interacts with tobacco constituents such as nicotine and NNAL, and may damage or kill cells. Key findings include:

• Data on menthol’s effects on biomarkers of smoke exposure, including nicotine/cotinine, CO (expired CO, blood carboxyhemoglobin), and some TSNAs are inconclusive.

• Menthol is a biologically active compound that may damage or kill cells.

• Menthol does not appear to alter the cytotoxic effects of tobacco smoke.

• Menthol reduces feelings of respiratory discomfort, but there are no corresponding physiological effects.

• The data regarding the effect of menthol on cardiovascular responses to cigarette smoke are inconclusive, however there is some evidence that smoking menthol cigarettes may produce worse cardiovascular effects as compared to nonmenthol cigarettes.

• Overall, the data regarding menthol cigarette smoke and cancer do not support a link between menthol cigarette smoke and increased risk of cancer, however there are some limited data that suggest possible menthol x gender x disease interactions.

Thus, based on the data reviewed in this paper, menthol cigarettes do not generally appear to be more harmful than nonmenthol cigarettes; both cigarettes produce significant negative effects on health outcomes, including respiratory disease, cardiovascular outcomes and cancer. However, there is some indication that menthol cigarettes may result in worse acute cardiovascular outcomes. In addition, there may be subgroups of smokers that may be more or less sensitive to the health effects of smoking cigarettes (e.g., race x gender x menthol interactions). It is interesting to note that although there were some indications that menthol smokers may be harmed more by cigarette smoking as compared to nonmenthol smokers, there were no indications of the converse.

## Competing interests

The author declares that they have no competing interests.

## Acknowedgement

Disclaimer: This article reflects the views of the author and should not be construed to represent FDA’s views or policies.

This article has been published as part of *Tobacco Induced Diseases* Volume 9 Supplement 1, 2011: Mentholated cigarettes and public health. Publication of this supplement has been supported by the Center for Tobacco Products, Food and Drug Administration. The full contents of the supplement are available online at http://www.tobaccoinduceddiseases.com/supplements/9/S1
